# Development Work in Swedish Eldercare: Resources for Trustworthy, Integrated Managerial Work During the COVID-19 Pandemic

**DOI:** 10.3389/fpubh.2022.864272

**Published:** 2022-06-30

**Authors:** Lotta Dellve, Anna Williamsson

**Affiliations:** ^1^Department of Sociology and Work Science, Faculty of Social Sciences, University of Gothenburg, Gothenburg, Sweden; ^2^Department of Biomedical Engineering and Health Systems, School of Chemistry, Biotechnology and Health, Royal Institute of Technology, Stockholm, Sweden; ^3^Production and Work Environment, Methodology, Textiles and Medical Technology, RISE Research Institutes of Sweden, Stockholm, Sweden

**Keywords:** change leadership, home care service, capability, managerial work practice, leadership, elder care, organizational improvement, organizational developments

## Abstract

The extensive needs for developments of eldercare addressing working conditions, care quality, influence, and safety was highlighted during the pandemic. This mixed-method study contribute with knowledge about capability-strengthening development work and its importance for trustworthy managerial work, before and during the COVID-19 pandemic. Questionnaire data and narratives from first-line managers immediately before (*n* = 284) and 16 months into the pandemic (*n* = 189), structured interviews with development leaders (*n* = 25), and documents were analyzed. The results identify different focuses of development work. Strategic-level development leaders focused the strengthening of old adults' capabilities. While operational-level leaders approached strengthening employees' capability. First-line managers' rating of their trustworthy managerial work decreased during the pandemic and was associated with their workload, development support and capability-strengthening projects focusing employees' resources. The study demonstrates the gap between strategic and the operational levels regarding understanding of capability set and needed resources for strengthening capabilities and trustworthy, integrated managerial work regarding safety, influence, and quality conditions for old adults and employees.

## Introduction

In Sweden and globally, current and future demographic data clearly indicate a growing population of older citizens in need of eldercare, combined with a smaller population of younger citizens in the labor market. The need for sustainable reform and organizational development that is both reliable and manage to maintain or strengthen the capabilities of the old adults, employees, and eldercare organizations has accordingly been advocated for several decades (1, 2). The COVID-19 pandemic has emphasized this need by exposing the insufficient resources allocated to eldercare and the poor trustworthiness in terms of poor quality, safety, and working conditions, as well as lack of influence among the old adults and employees (3). This study identifies the focus of and conditions for ongoing development work at the strategic and operational levels, noting the importance of this work for trustworthy operational management work.

### Strengthening Individual and Organizational Capabilities in Eldercare

The capability approach (4, 5) refers to the ability and resources needed to live a life and work in a way one values, and to the conditions that strengthen that ability. An individual's capability is determined by his or her access to the resources and ability needed to convert resources into personal goals (5). Organizational capability refers to an organization's ability to implement management practices based on its resources and preferences (6). Resources may refer to general and specific conditions and to organizations' management strategies. The configurations of resources determine the frame of opportunities available to realize chosen goals. The concept thus offers an analytical tool with which to understand the conditions determining individuals' and organizations' ability to realize goals by converting resources.

A capability approach to eldercare is vital if we are to understand how the way we organize and strengthen the conditions of eldercare promotes the opportunity to realize the preferences and values of the old adults, employees, and the eldercare organization. This refers to the conditions determining: (a) old people's capability to use their resources to make choices about their living conditions; (b) employees' capability to use their resources at work; and (c) the organization's capability to implement its strategies and provide good service during crises.

Capability-strengthening development projects may have different focus, grounds and conditions. The strengthening of *older adults*' *capabilities*, which addresses their opportunities to influence and participate in decisions concerning their lives, is regulated by law in Sweden. However, using opportunities of capabilities require influence over every-day decisions, abilities and skills, e.g. eHealth literacy (7). The strengthening of *employees*' *capabilities* addresses their working conditions and the resources needed to conduct their work, such as influence, competence, and health. This strengthening is needed, with or without a pandemic situation, since eldercare employees frequently face higher risk exposures in their working environment, experience more occupational disorders, take more sick leave, and display earlier retirement behavior (1). While employees in other sectors in Sweden have recently faced decreased work demands (8), eldercare work still entails a sector with increasing work demands (9, 10). In countries with public eldercare, attempts have been made to strengthen *organizational capabilities* using different governance mechanisms to meet current and expected demands while upholding appropriate quality and efficiency (2). Also, new forms of ownership and organization have been trialed (11). However, multiple development projects being managed simultaneously without coordination may explain the poor results of such efforts in terms of capability and trustworthy developments (12).

### Supportive and Hindering Conditions for Trustworthy Implementation

A systematic review of the barriers to and facilitators of welfare technology implementation in eldercare has identified the capacity of the old adults, employees, and eldercare organizations as a recurrent factor affecting implementation success (13). The work and leadership of first-line managers (FLMs) has been crucial for sustainable work by employees during the COVID-19 pandemic (3, 14). Earlier studies show that FLMs' efforts to link organizational levels to integrate perspectives and serve core purposes have achieved some success in terms of sustainable development in public care (15, 16). This puts high demands on managers and requires supportive organizational conditions and resources (17). However, poor vertical alignment, excessive managerial workload, and poor functional support have been identified as key obstacles to successful development work among FLMs (18). In fact, one central hindrance to the development and implementation of needed changes in healthcare is deficient practical operationalization of strategies (19–21) and a lack of vertical alignment within organizations (22). In eldercare, studies have reported challenges regarding vertical alignment due to insufficient resources and followup in the implementation process (20, 23, 24). To support organizational capability, development leaders (DLs) and change-supporting functions at different organizational levels can support the conversion of resources generated in development projects into improved practice at operational levels (25–27), i.e., trustworthy management. Methodological and contextual knowledge along with legitimacy in communicating and negotiating between organizational levels are key features of these roles (21, 28). There is little knowledge of the work and importance of DLs in managing necessary development work of different kinds during pandemic crises.

According to systems theory, the strengthening of resources necessary for capabilities needs to be informed by integrated perspectives that capture key conditions for development work and various resources at all system levels (29). This is supported by recent case studies in eldercare showing that resources for and perspectives on capability must be addressed at all organizational levels in order to support improvement and development work (13, 24, 30). A logic suggested to be more successful for public health and social service is to strengthen integrated values (31) in order to support the sustainable development of the capabilities of elders, employees, and eldercare organizations (32). The interaction between development strategies at the strategic and operational management levels can explain the success and sustainability of development work (33).

### Aim

This study contribute knowledge about capability-strengthening development work in eldercare and its importance for trustworthy managerial work, before and during the COVID-19 pandemic. The following research questions were addressed: Who's capabilities are focused on and who is initiator, driver and active participant in the development project? What capability-strengthening projects are actively conducted in eldercare units? How are FLMs' trustworthy managerial work associated with the development projects, their workload and development support?

## Materials and Methods

### Study Design

A mixed-method design, with parallel qualitative and quantitative data-collection and analysis, was chosen to explore, identify and assess ongoing development work in municipal eldercare organizations. The research questions were answered using data from semi-structured interviews with DLs, organizational documents, and questionnaire data with FLMs. Ethical approval was given by the Regional Ethics Committee (Dnr: 2019-02934).

### Setting

Eldercare in Sweden is provided by a comprehensive public care system covering all citizens and funded by tax revenue. Various laws and regulations cover older people's choice of care and living arrangements, i.e., the Social Service Act (2001:453), Healthcare Act (2017:30), and Freedom of Choice Act (2008:962), and appropriate working conditions, i.e., the Work Environment Act (1977:1160) (34). These are followed up within eldercare organizations and by government authorities such as the Health and Social Care Inspectorate. The municipalities have the responsibility for providing good and safe eldercare for their citizens. Since the 1990s, the state has financially supported development work, through The National Board of Health and Welfare and by means of several national training initiatives. The purpose of these programs is to subsidize and incentivize, for example, development work focusing quality improvements and measures to increase the competence of the eldercare workforce. Depending on their size and economic conditions, municipalities have central development units supporting development work within eldercare. Where applicable, municipalities have assigned local DLs to work closely with operational eldercare management. FLMs are responsible for the service quality, personnel, working conditions, efficiency, and budget at their units; as such, they are responsible for integrating and putting into practice multiple values, perspectives, and policies of eldercare.

### Study Sample

First, a questionnaire was distributed to eldercare FLMs in a random selection of 33 of Sweden's 290 municipalities. The selected municipalities were geographically situated in the northern, southern, eastern, and western parts of the country. These municipalities came from eight of the nine categories of municipalities, based on structural parameters such as population and commuting patterns (35). The randomly selected municipalities did not include any large cities, so one of Sweden's three large cities was also selected—for practical reasons, the nearest one. In the selected municipalities, all eldercare FLMs were identified through websites and direct contact with administrators. In the large city, a list of all FLMs, including their email addresses, was provided by the municipal administration.

The questionnaire was distributed via personal email addresses in the winter of 2019–2020 (T1), to 548 FLMs understood to be FLMs. 284 agreed to participate (response rate 52%). The followup (T2) questionnaire was sent in May–June 2021 to 472 eldercare managers understood to be FLMs. Of the 206 managers who completed the questionnaire, 189 were actually FLMs (response rate, 40%).

Second, during March–June 2021 14 municipalities varying in size, proportion of old adults living in assisted livings, and geographic location were selected for deeper investigation. In these municipalities, documentation of development work strategies was obtained and the support functions for development work were identified. These support functions were identified in all 14 municipalities, in some at multiple organizational levels. These development-supporting roles (henceforth, development leaders [DLs]) in the municipalities' elder care were: a) appointed DLs at different organizational levels, b) development managers responsible for development within a certain part of social care, or c) project managers, DLs, or care professionals assigned responsibility for certain development projects. There were DLs placed in and supporting strategic management (in eight municipalities), DLs placed in and supporting operational management (in five municipalities), and DLs placed at the strategic level but supporting operational management (in five municipalities). In one municipality, there was no specially appointed development support at any management level, and in another municipality, the responsibility for development was given to different operational managers, depending on the project type, but with no special development support.

The interviewed *strategic-level DLs* (*n* = 15) had positions closer to the political level. *Operational-level DLs* (*n* = 9) either had a position at the same hierarchical level as the second line manager or worked in close communication and collaboration with the operational level. Four out of the 24 interviewees were men. 40% had manager positions. 29% had worked up to one year, 42% 1–3 years, and 29% 4–8 years in their position. Their backgrounds varied from several years of working in different positions within the same or another municipality in different social care fields, to backgrounds in behavioral or political science and industrial managerial work.

### Data Collection

#### Interviews With Development Leaders

Semi-structured interviews were conducted with DLs at the strategic and operational levels regarding strategies, the development of organizational preconditions, and the rated state of the development work (i.e., in terms of focus, initiative, drive, and collaboration). The interviewees were asked to rate the actual state of the work, not the vision for it. The data were analyzed according to the interviewees' closeness to the strategic or operational level. Most interviewees found this rating quite difficult, but the rating of the most common to the third most common alternatives was easier; ranks 1–3 are therefore considered most reliable.

#### Questionnaire

The web-based questionnaire included items capturing managerial conditions, supporting resources, and improvement work. In this study, the following variables were selected to answer the research questions.

##### FLMs' Development Conditions

FLMs' *development-supporting resources* were assessed using the item: “I have trusting cooperation with resource functions (i.e., developers, improvement managers, or the equivalent) in work on organizational improvement.” FLMs' *excessive workload* was assessed using an index of four items (Cronbach's alpha = 0.83): working overtime, being unable to rest from work, not having time for all the work to be done, and private/family life suffering due to managerial responsibility. The items could be responded to on a scale ranging from (1) “No, not at all” to (5) “Yes, to a high degree.” Both these variables came from the Gothenburg Manager Stress Inventory (36).

##### Active Development Work at Eldercare Units

The question capturing current organizational development projects with a strategic focus for the purpose of strengthening resources needed for capability improvement was developed through interviews with 80 strategic-level managers (forthcoming). The question started: “At your unit in the past year, have you driven or actively participated in development work/projects regarding,” followed by a list of projects that could be responded to on a scale ranging from (1) “No, not at all” to (5) “Yes, to a very high degree.” The examples of development projects were grouped according to the main focus of the resources in the development project, i.e., whether they were directed toward strengthening *older people*'*s, employees*', or *organizational capabilities* (see **Table 3**).

##### Trustworthy, Integrated Managerial Work

An index of six items assessing systematic occupational health and safety management practice was used (37). The question started: “Are you satisfied with your opportunities to fulfill your managerial responsibilities, in a trustworthy and safe manner, in the following areas,” followed by *daily work, influence of the old adults, care quality, safety, employee influence*, and *working conditions*. The items could be rated on a scale ranging from (1) “No, not at all” to (5) “Yes, to a very high degree.” The respondent could also respond: “Don't know/not relevant.” The internal consistency was high (Cronbach's alpha = 0.90).

### Documents

In the 14 municipalities selected for deeper investigation, certain organizational documents were requested following the interviews, to complement and validate interview data. These were documents on development and improvement work concerning organizational vision, strategies, and arrangements to support preconditions for improvement work at the operational level.

### Analysis

The interviews and organizational documents were analyzed qualitatively. All interviews were transcribed verbatim and analyzed using thematic analysis (38). The structured questions in the interviews were analyzed according to the percentages of interviewees rating certain alternatives as the most emphasized/common/important, and other alternatives as the second most emphasized/common/important, and so on. The document analysis was conducted from the same basis as the interview analysis for both data sources to verify each other.

Data from the questionnaires administered to first-line managers were analyzed using: (a) descriptive analysis (m, sd, %) regarding driving or actively participating in development work; (b) explanatory analysis using a series of forward stepwise regressions to select the most statistically important active development work/projects in each focus area (i.e., older people's/employees'/organizational capability) of trustworthy managerial work; and (c) theoretical stepwise regression models assessing the importance of additional focus areas in active development work and the importance of FLMs' development conditions.

## Results

**Figure 1:**
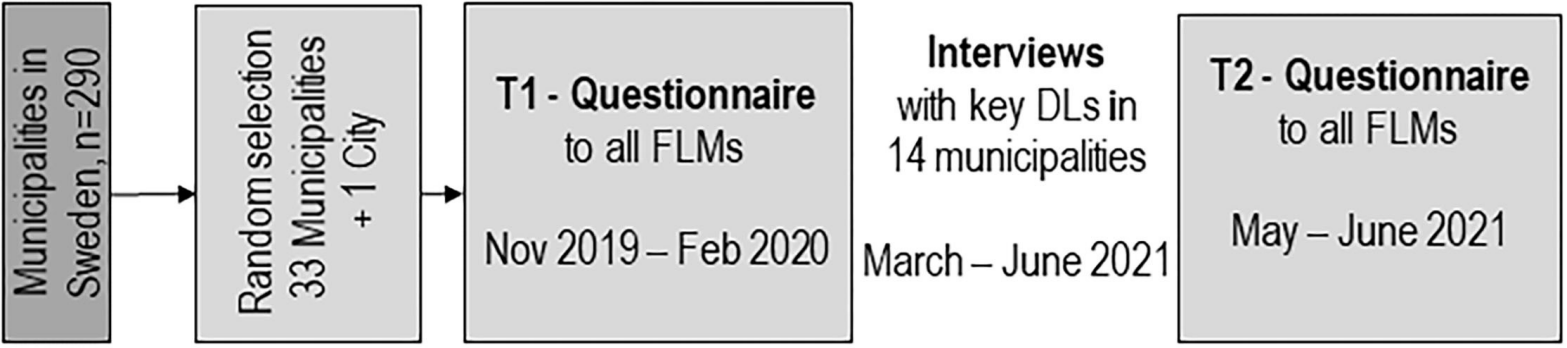
Overview of the data-collection and study-populations.

An overview of the data-collection phases and study-populations included to form the base for the analysis of capability-strengthening development work in eldercare are described in Figure 1 and Table 1. The result of the analysis are described sequentially. First, the development work of DLs at the strategic and operational levels regarding focus, initiators, drivers and active participants are described. Second, the various operational development work conducted at eldercare units, including supportive conditions and impact on trustworthy, integrated managerial work are presented, based on questionnaire data answered by FLMs.

**Table 1:** Demographic information of responding first line managers (FLMs).

	**T1**	**T2**
FLM, responding/selected, *n* (%)	284/548 (52%)	189/472 (40%)
Municipalities, responding/selected *n* (%)	34/34 (100%)	27/34 (79%)
Female FLMs, *n* (%)	257 (90%)	166 (88%)
Age: 18–34 years, *n* (%)	18 (6%)	14 (7%)
35–54 years	168 (60%)	113 (60%)
55–67 years	98 (35%)	62 (33%)
Experience as manager, yrs (m/md)	13/12	12/11
Number of employees (m/md)	31/30	35/32

### Development Work Among Development Leaders at the Strategic and Operational Levels

Development work was described by DLs as project driven and mainly applying a top–down perspective. The DLs perceived development work as having a poor likelihood of success, telling of few activities and measures to follow up or evaluate development work. This was seen also in documents only mirroring strategies and vision of development work, and not presenting any development activities, results, follow-up on development projects or organization's best practice. The poor operational-level implementation was understood by DLs to be affected by employees and FLMs who lacked competence, did not understand the core principles of implementation work, and sometimes even lacked basic healthcare competence to implement, for example, new hygiene routines. There was also talk of a culture of concentrating only on the core tasks of eldercare and of exhaustion and a fear of change following years of organizational restructurings.

“I think a lot depend on top management or so, what it looks like, what they push for and see as essential. But of course, if it goes on for many years and you cut down on…. Opportunities for learning and developing…you get a culture among staff that you stick to doing what you must with the least effort…yeah” (strategic level developer in a smaller municipality).

In several municipalities, project ownership was seldom implemented properly from the strategic to operational levels, as intended. One example was cited by a strategic-level interviewee who identified a bottleneck in the transition between the development unit and the homecare units. The operational level lacked sufficient resources to take over projects, which were consequently returned to the development unit, so the homecare units could not be accounted for in the implementation phase. Another example was a project in a mid-sized municipality in which grocery shopping by the old adults was digitalized in order to: (a) increase the influence of the old adults on the shopping; (b) minimize the manual handling of papers between the older people's homes and the homecare office; and (c) minimize the time spent on administration between visits by homecare staff. Instead of following the project plan for the implementation phase and following the instructions for using the iPads with the old adult, staff interpreted the instructions in their own ways. They continued to take food orders on paper, accumulating the shopping lists and submitting all the orders at once from the homecare office computer. This increased the administrative burden on staff between visits, so the goal of increasing the influence and involvement of the old adults was not attained. Another strategic level developer from a smaller municipality put it this way:

“And then you think that within home care or elder care, you get a bit scared too. Because when facing a new… “Oh my God, I don't know this computer system!” It's a challenge, to get people to learn new things. That we need to work differently. We have been working now… for like 30 years with these papers. But now we are facing digitalization. And then we need to catch up, you know.”

### Focus, Initiative, and Drive

Strategic-level DLs had a clear client perspective, and the development work was seen as a means of reaching the goals of the social or elder care political committee. Their work emphasized the influence and participation of the old adults. DLs working closely with the operational level focused more on development projects concerning employees' working conditions or old adults' security, and applied the perspective that employee well-being was a precondition for caring for the old adults. This was reflected in their rated *focus of development work* in eldercare. At the operational level, most rated “employee working conditions” as the first priority, vs. the strategic level, where most rated “old adults' influence on and participation in their own care” as the first priority (Table 2). About 60% of strategic-level respondents rated “employee working conditions” as the second priority. About 30% of operational-level respondents rated “old adults' security” as the first priority, while about 75% at the operational level rated “old adults' security” and “old adults' influence on and participation in their own care” as the second priority.

**Table 2:** Focus, initiative, driver, and contribution for development projects in eldercare, rated by developments leaders.

**Question**	**Rating alternatives**	**Strategic (*N* = 15)** **n Md (%)**	**Operational (*N* = 9)** **n Md (%)**
**Focus**	Influence of old adults	**15** 1 (53%)	**9** 3 (44%)
	Working conditions	**15** 2 (60%)	**8** 1 (56%)
	Employee influence	**15** 3 (47%)	**8** 4 (67%)
	Safety issues	**15** 3 (33%)	**9** 2 (44%)
**Initiator**	Strategic mgmt./politics	**15** 1 (73%)	**9** 1 (56%)
	DLs/change agents	**14** 2 (60%)	**7** 3 (33%)
	FLMs	**14** 3 (33%)	**9** 2 (56%)
	Assistant nurses	**14** 4 (53%)	**8** 4–5 (22, 22%)^*^
	Old adults	**14** 5 (20%)	**9** 5 (22%)
	Relatives	**14** 5–6 (33, 20%)^*^	**9** 5 (22%)
	HR	**15** 7 (67%)	**8** 4–5 (22, 22%)^*^
**Driver**	Strategic level	**14** 1–2 (47, 20%)^*^	**8** 1–2 (44, 33%)^*^
	DL	**15** 2 (27%)	**8** 1–2 (44, 11%)^*^
	Operational level	**13** 3 (13%)	**8** 2–3 (44, 11%)^*^
	FLMs supported by DLs	**14** 3 (40%)	**8** 3 (44%)
	Employees supported by DLs	**14** 5 (53%)	**6** 5 (44%)
**Active participant**	FLMs	**15** 1 (73%)	**9** 1 (78%)
	Assistant nurses	**14** 2 (53%)	**9** 3 (67%)
	HR/other support function	**15** 3 (33%)	**8** 2 (56%)
	Old adults	**15** 4 (53%)	**8** 4 (67%)
	Relatives	**14** 5 (60%)	**8** 5 (67%)

*Median rating (Md), number of interviewees (n), and percentage of total number of interviewees' (N) ratings according to median rating (%). Rating: 1 = most common, 2 = second most common, etc*.

* *Median rating of the alternative lying between two values, hence two percentages for the total number of interviewees*.

*Bold only means to separate the numbers*.

Regarding *stakeholders who demand or initiate development in eldercare*, all respondents rated “strategic management or politics” as the stakeholder most commonly initiating development efforts. Strategic-level respondents rated DLs as the stakeholder the second most likely to initiate development efforts, while operational-level respondents rated FLMs as the second most likely. In interviews, several DLs expressed a desire for more involved old people, their relatives, and operational-level management and employees when it came to generating ideas for development efforts, but they also said that the operational level lacked the conditions to prioritize such efforts.

Some strategic-level interviewees spoke of how they usually (before the pandemic) had dialogue meetings with citizens/the old adults and their relatives, and several wished that the old adults and their relatives would be more involved in generating ideas for development efforts. All DLs had optimistic, even naïve, beliefs about the importance of their contributions to the development work, rating “the strategic level” or DLs as the most common *drivers of development* in eldercare. All interviewees highlighted the importance of engaging the operational level in development, and many said that FLMs should be the key actors in driving change. Again, and as seen in the focus of the document narratives, the results indicate that this seemed to be more of a vision than the reality. Concerning *stakeholders actively participating in eldercare development*, interviewees at both levels agreed that FLMs were the most active contributors to development efforts; the next most active were “human resources or other supporting resource” or “assistant nurses.”

### First-Line Managers Development Work

Table 3 presents FLMs' assessments of their conditions for development work and their ability to conduct trustworthy, integrated managerial work. The development support increased during the pandemic. At T2, half of the FLMs (51%) received trustworthy organizational support from DLs. The managerial work on safety of the old adults was the lowest at T1 and had improved at T2. Almost all other aspects (except employee influence) of managerial work responsibilities, including summed trustworthy, integrated managerial work, had decreased.

**Table 3:** FLM-assessed conditions for development and ability to conduct trustworthy, integrated managerial work before COVID-19 (T1) and 16 months into the pandemic (T2).

	**T1 m (sd)**	**T2 m (sd)**
**Development conditions**		
Development support	3.41 (1.21)	3.61 (1.23) ^a^
Excessive workload	3.14 (0.99)	3.30 (1.12)
**Trustworthy, integrated managerial work**	3.65 (0.41)	3.38 (0.81) ^b^
– Safety of the old adults	2.19 (0.71)	3.33 (0.99)^a^
– Influence of the old adult	3.61 (0.93)	3.20 (1.05)^b^
– Working conditions	3.76 (0.87)	3.39 (0.95)^b^
– Employee influence	3.84 (0.90)	3.67 (0.94)
– Care quality	3.64 (0.89)	3.33 (1.06)^b^
– Daily work	3.67 (0.94)	3.28 (0.94)^b^

a *Increased T1–T2, Wilcoxon signed-rank test, p < 0.05*.

b *Decreased T1–T2, Wilcoxon signed-rank test, p < 0.05*.

#### Operational Development Work

A range of development projects was more or less actively driven at the operational level, with the intention of strengthening resources to bolster the capabilities of old adults, employees, and the eldercare organization. Table 4 shows the degree of activity of development projects at T1 and T2. The most common development projects with a *focus on the old adults* concerned “digitization to improve eldercare quality” (active projects at 48% of the units at T1) and “increased influence of the old adults on operational decision-making” (active projects at 32% of the units at T1). The most common projects with an *employee focus* concerned “increased employee influence on operational decision-making” (active projects at 52% of the units at T1) and “strengthening competence” (active projects at 49% of the units at T1). Projects with an *organization focus* concerned “digitization to improve followup of a) care quality, b) economic aspects, and c) staff planning.” These were common and active projects, with 65% of units actively involved in digitization related to economic aspects, 56% involved in digitization related to care quality, and 47% involved in digitization related to staff planning.

**Table 4:** Active development work at eldercare units, for the purpose of strengthening resources for the capabilities of the old adults, employees, and eldercare organizations, and correlation with FLMs' trustworthy, integrated managerial work; p > 0.1 considered non-significant (ns).

	**Descriptives**		**Stepwise regressions**	
	**m (SD)**		**Trustworthy, integrated managerial work** ***r***^2^***/r***^2^ adj.^**^, Beta (***p***-value)	
**Development focusing on:**	**T1**	**T2**	**T1**	**T2**
**The old adults (Cronbach's alpha 0.75 resp. 0.61)**	**2.37 (0.88)**	**2.65 (0.89)**	**0.19/0.18**	**0.22/0.20**
Digitization to strengthen eldercare quality	3.30 (1.36)	3.12 (1.29)	ns	ns
Increase influence of the old adults on operational decision-making	2.80 (1.24)	2.41 (1.12)	0.22 (0.00)	0.17 (0.03)
Develop models of businesses driven by employees and/or old adults (e.g., intrapreneurship and social entrepreneurship)	1.78 (1.28)	2.34 (1.13)	ns	0.22 (0.00)
**Employees (Cronbach's alpha 0,74 resp. 0,75)**	**3.06 (0.90)**	**2.84 (0.82)**	**0.25/0.24**	**0.30/0.29**
Digitization to decrease employee workload	3.19 (1.42)	2.95 (1.19)	ns	ns
Increase employee influence on operational decision-making	3.29 (1.17)	3.02 (1.24)	0.18 (0.00)	0.23 (0.00)
Technical development to decrease workload	2.67 (1.39)	2.5 (1.27)	ns	ns
Strengthen knowledge and competence to handle work	3.25 (1.13)	3.12 (1.08)	0.19 (0.00)	ns
Supervision or mentorship of newly recruited	2.99 (1.23)	2.70 (1.2)	ns	0.22 (0.00)
**Organization (Cronbach's alpha 0.78 resp. 0.75)**	**2.62 (0.84)**	**2.58 (1.04)**	**0.14/0.13**	**0.15/0.14**
Digitization to improve followup of care quality	2.63 (0.96)	2.61 (1.23)	ns	0.27 (0.00)
Digitization to improve followup of economic aspects	2.76 (0.98)^*^	2.33 (1.19)	−0.22 (0.00)	ns
Digitization to improve staff planning	2.47 (1.08)^*^	2.77 (1.35)	ns	ns

*ns = p > 0,05*.

* *Not in the city*.

** *r^2^/r^2^ adj. from stepwise regression for each resource dimension; variables were excluded if p > 0.1*.

*Bold only means to separate the numbers*.

A series of forward stepwise regressions, one for each development focus, identified the development work with the greatest impact on trustworthy, integrated managerial work (Table 4). The highest explained variance of managerial work (*r*^2^ = 0.30) was found at T2 in employee-focused development efforts, specifically projects addressing increased employee influence on operational decision-making and the introduction/mentorship of new employees. The projects focusing on the old adults explained about 20% of the variance in managerial work, specifically as regards increased influence of the old adults on operational decision-making at T1 and developing models of business driven by the employees and/or old adults (e.g., intrapreneurship and social entrepreneurship) at T2. Digitization of the followup of care quality explained 10–15% of the variance in trustworthy managerial work.

#### Importance of Development Work for Trustworthy, Integrated Managerial Work

In stepwise models 1–4, the contribution of operational development work to strengthening FLMs' managerial work at T2 was modeled (Table 5). Model 1 showed the importance of FLMs' organizational support from DLs, and the importance of this support was further examined in the following models. Models 2–4 included the operational development work focusing on the capabilities of eldercare organizations, the old adults, and finally employees. FLMs' development support explained 6% of the variation. The development work explained 23% of the variation, most strongly for the employee-focused development work. The final model also included the main obstacle—FLMs' excessive workload. This had some impact and explained an additional 15% of the variance in trustworthy managerial work. The same modeling was conducted for T1 with about the same pattern of associations: the employee-focused development work and FLMs' excessive workload had the strongest associations with trustworthy managerial work, while FLMs' development support, a focus on the old adults, and an organizational focus had weaker associations. The final model explained 36% of the variance at T1 and 44% at T2.

**Table 5:** Stepwise models of the importance of FLMs' development conditions and implemented resources for trustworthy, integrated managerial work at T2.

	**Trustworthy, integrated managerial work** **Beta (*p*-value)**				
	**Model 1**	**Model 2**	**Model 3**	**Model 4**	**Model 5**
**Development support**	0.17 (0.03)	0.27 (0.00)	0.12 (0.10)	0.12 (0.09)	0.09 (0.14)
**Development work**					
Organizational focus		0.13 (0.08)	0.18 (0.04)	0.17 (0.04)	0.09 (0.14)
Focus on the old adults			0.25 (0.02)	0.08 (0.55)	0.06 (0.60)
Employee focus				0.23 (0.04)	0.26 (0.01)
**Excessive workload**					−0.28 (0.00)
Intercept	2.73	2.17	1.86	1.63	2.59
*R* ^2^	0.07	0.22	0.29	0.34	0.48
*R*^2^ adj.	0.06	0.20	0.25	0.29	0.44

## Discussion

The aim of this study was to build knowledge of development work to strengthen resources supporting the capabilities of the old adults, employees, and eldercare organizations. This aim was operationalized by addressing three research questions concerning: the development work targeted by DLs at the strategic and operational levels in eldercare; the operational development work at eldercare units and its importance for FLMs' trustworthy, integrated managerial work; and the FLMs' development conditions in terms of workload, development support, and the association with their trustworthy, integrated managerial work.

Answering the first questions, the study identified multiple ongoing development projects in eldercare with the objective of strengthening resources for eldercare organizations, the old adults, and employees. However, active driving of and participation in these projects at operational levels were limited. Strategic-level DLs reported the greatest emphasis on the influence of the old adults on eldercare development, while operational-level DLs reported the greatest emphasis on employee working conditions, in line with most work-unit activity being projects with an employee focus. There is reason to believe that, from an FLM perspective, employee working conditions are seen as a precondition for offering trustworthy eldercare, both before [see, e.g., (15, 17)] and here during the pandemic.

Unsurprisingly, FLMs' managerial work on the safety of the old adults increased during the pandemic, apparently at the expense of almost all other aspects of trustworthy, integrated managerial work (i.e. quality of care, influence of the old adults, daily work and working conditions). Further, also all kinds of development work decreased during the pandemic except digitization to improve staff planning which was increased. FLMs' development support also increased somewhat during the pandemic, but remained moderate. This was confirmed by operational-level DLs, who stated that development work co-driven by DLs and FLMs was not as common as at the strategic level or as purely DL-driven development work. Still, DLs highlighted FLMs as the most active drivers of eldercare development work. The development support had some positive impact, while excessive workload obviously had some negative impact. Summed up, the development conditions had some importance, but operational-level employee-focused development work was the most important for FLMs' perception of performing trustworthy, integrated managerial work. Despite operational development work being somewhat limited 16 months into the pandemic, ongoing development work seemed especially important for FLMs' perceptions of their own managerial work during the pandemic.

The findings suggest that there is synergy between strategic-level development work, operational-level development work, and FLMs' personal ratings of their resources for performing managerial work in a trustworthy, integrated way. These kinds of synergies were earlier discussed in relation to the capability set concept (5) and the crafting of sustainable work through the development of personal resources, translated to employees' work ability and collaborative work crafting (39). Thus, active participation in development work in one's own unit seems to create learning, strengthening the FLM's ability to contribute to overall organizational capability. The capability set may also entail difficulties for managers struggling to convert resources due to their excessive workload. Support for this interpretation can be found in studies showing associations between managers' work performance and their stressors and excessive workload (18, 40). Other studies of healthcare have identified stressors in terms of hard control and top-management demands impinging on the work of FLMs (28, 41). This study builds knowledge of the importance of excessive workload for the capacity to perform trustworthy, integrated managerial work that integrates important capabilities of eldercare service of value for the old adults, employees, and the eldercare organization.

When elaborating on the answer to the last research question, the findings also raise questions regarding the development support in terms of content of the resource. Multiple, parallel projects (12) and limited development support likely constrain FLM potential to be a resource in strengthening organizational capability. Over the last four decades, various models, or best practices, have emerged to fit and strengthen resources in different contexts (42). DLs are commonly part of the facilitating processes when implementing change. As mentioned above, the present results as well as results of previous healthcare change management research (27) tell of high expectations of FLMs as change drivers in organizational development. However, the FLM role is restricted to a certain unit in an organizational hierarchy. Development support from DLs or change agents has been stressed as important in order to ease the burden on FLMs as well as to take responsibility for aligning strategy and operationalization when driving change (27, 28). Is the insufficient development support for FLMs studied here based on a lack of development-supporting resources in the municipalities, or is it because the supporting resources do not meet the needs of the FLMs or help them in aligning operations with strategy?

Today's poor conditions for capability among old adults and the external pressure to develop eldercare due to ongoing demographic shifts points toward new ways of organizing as well as toward digitization and the implementation of welfare technology. Critical factors seen to affect the implementation of new technology are, besides capacity and aHealth literacy, as mentioned in section 1.2, attitudes and values in the eldercare workforce (13). The present study also identifies the diverse importance related to aim and focus of digitalized developments, i.e. digitization to increase influence vs. followup of economic aspects. The further development of eldercare also depends on the sustainable work of FLMs who have the supporting resources in place to ease their workload and increase time spent on driving change.

This research has certain limitations and strengths that merit consideration. Strengths that made the interpretation of findings more valid were: (a) the comparatively wide-ranging sampling across Sweden, including a random selection of municipal eldercare organizations; (b) the combination of qualitative and quantitative data; and (c) the stepwise systematic data collection using validated measures. Limitations of the sample were (a) the poor response rate (56 and 40% at T1 and T2, respectively), and (b) the high FLM turnover, limiting the ability to follow up individual FLM responses.

## Conclusions

Development work regarding the safety of the old adults and digitization of staff planning increased during the pandemic, while other kinds and aspects of development work decreased or remained at the same level. Further, the study confirms the previously noted abundance of development work being conducted in eldercare in Sweden. The focus of this development work differed depending on where in the eldercare organization one asked questions about it. Eldercare DLs at the strategic level told of prioritizing the influence of the old adults, while DLs working closer to the operational level told of prioritizing employee influence and working conditions. Most development work was initiated and driven by the strategic level, despite the strong conviction that FLMs ought to be the best change drivers in implementation. However, excessive workload and moderate development support hindered FLMs trustworthy managerial work. They worked most active with approaching capability-strengthening projects focusing employees' resources. This focus was also most strongly associated with their rated performance of trustworthy, integrated managerial work. While the capability set for other projects seem not to be at place. These findings call for the further investigation of a suitable development support functions, both to ease FLMs excessive workload and to increase their opportunity for active approaching capability-strengthening developments in eldercare.

## Data Availability Statement

The data supporting the conclusions of this article will be made available by the authors, without undue reservation.

## Ethics Statement

The studies involving human participants were reviewed and approved by the Regional Ethics Committee (Dnr: 2019-02934). The patients/participants provided their written informed consent to participate in this study.

## Author Contributions

LD and AW have both participated in the conception of the study, study design, and interpretation of the overall analysis. LD has managed the distribution of the survey, the quantitative analysis of the survey results, and drafted the manuscript. AW has performed the interviews, analysis of interviews and documents, complemented with texts, and critically revised the manuscript. Both authors contributed to the article and approved the submitted version.

## Funding

This work was funded from Swedish Research Council for Health, Working-Life and Welfare (Forte Dnr 2020-01579).

## Conflict of Interest

The authors declare that the research was conducted in the absence of any commercial or financial relationships that could be construed as a potential conflict of interest.

## Publisher's Note

All claims expressed in this article are solely those of the authors and do not necessarily represent those of their affiliated organizations, or those of the publisher, the editors and the reviewers. Any product that may be evaluated in this article, or claim that may be made by its manufacturer, is not guaranteed or endorsed by the publisher.
